# Molecular Determinants of Osseointegration in Implant-Supported Prostheses: A Narrative Review of Gene Expression Signatures, Signaling Pathways, and Bioinformatic Insights

**DOI:** 10.7759/cureus.112219

**Published:** 2026-07-07

**Authors:** Cristian Villamizar, Joanith Medina Barreto, Sara L Ascanio Lara, Astrid Gamez Vicuña, Jessica Tokatli, Estela E Lopez Alas, Luisana S Rodriguez

**Affiliations:** 1 Dentistry, Universidad Santo Tomás, Bucaramanga, COL; 2 Dentistry, Universidad de Carabobo, Valencia, VEN; 3 Dentistry, Universidad del Magdalena, Santa Marta, COL; 4 Dentistry, Universidad Nacional Experimental de los Llanos Centrales Rómulo Gallegos, San Juan de los Morros, VEN; 5 Dentistry, Universidad Nororiental Privada Gran Mariscal de Ayacucho, Barcelona, VEN; 6 Dentistry, Universidad Del Salvador, San Salvador, SLV; 7 General Dentistry, Boston University School of Medicine, Boston, USA

**Keywords:** bioinformatics, dental implants, gene expression, hub genes, implant-supported prostheses, osseointegration, osteogenic signaling pathways, peri-implant tissues, predictive biomarkers, transcriptomics

## Abstract

Osseointegration is a biologically complex process that determines the long-term success of implant-supported prostheses. Advances in molecular biology have shown that gene expression programs and signaling networks, rather than mechanical fixation alone, govern bone healing and implant integration. Yet, these molecular determinants are still seldom used to guide clinical decisions, which continue to rely on mechanical and histologic assessments. This narrative review addresses that gap by analyzing the molecular determinants of osseointegration through five connected perspectives: the cellular cascade that follows implant placement; the principal osteogenic signaling pathways (bone morphogenetic protein (BMP), Wnt/β-catenin, nuclear factor kappa B (NF-κB), and Runt-related transcription factor 2 (RUNX2)); transcriptomic signatures across the inflammatory, repair, and remodeling phases; bioinformatic gene regulatory and protein-protein interaction networks associated with implant success or failure; and the influence of implant surface properties on the molecular response. Across these perspectives, osteogenic and angiogenic genes such as RUNX2, collagen type I alpha 1 chain (COL1A1), bone gamma-carboxyglutamate protein (BGLAP), and vascular endothelial growth factor A (VEGFA) are consistently linked to successful integration, whereas sustained inflammatory and osteoclastogenic signatures such as IL6, tumor necrosis factor (TNF), matrix metalloproteinase-9 (MMP9), and nuclear factor of kappa light polypeptide gene enhancer in B-cells 1 (NFKB1) characterize failure. The review then considers how this knowledge may be translated into gene expression-based biomarkers, peri-implant crevicular fluid monitoring, patient risk stratification, and precision implant therapy, and identifies the main barriers to clinical adoption of an emerging implantogenomics framework.

## Introduction and background

Osseointegration is the biological foundation of implant-supported prosthetic rehabilitation. Brånemark originally defined it as a direct structural and functional connection between living bone and the surface of a load-bearing implant [1]. For decades, the concept was assessed primarily by histologic bone-to-implant contact and clinical stability, but it is now understood as a sequence of cellular and molecular events governed by coordinated gene expression programs that regulate inflammation, cell recruitment, osteogenesis, and remodeling [2].

This distinction matters clinically. Implant survival, meaning the fixture remains in place, is not the same as implant success, which reflects healthy, stable integration over time [3]. The interval between primary mechanical stability and secondary biological fixation is largely invisible to routine examination, yet it is the period of most intense molecular activity and the window in which integration is won or lost. Understanding the genes and pathways active during this phase is therefore central to improving the predictability of treatment and to the emerging goal of precision implant dentistry [4].

Several concepts recur throughout this review and are defined here for readers who are not specialists in molecular medicine. Transcriptomics is the study of the full set of RNA transcripts that a tissue expresses at a given moment, revealing the genes that are active during healing [5]. Gene regulatory networks describe how transcription factors and their target genes interact to coordinate that activity [6]. Bioinformatic analysis applies computational tools to these large datasets to identify the genes, pathways, and highly connected hub molecules that drive a biological outcome [7]. Applied to implant biology, these approaches shift the assessment of osseointegration from what is visible histologically to the molecular programs that determine whether bone-to-implant contact becomes stable and durable.

This narrative review integrates current molecular and bioinformatic evidence on osseointegration across five connected themes: the cellular cascade from implant placement to bone-implant contact; the principal osteogenic signaling pathways; transcriptomic signatures across the stages of healing; bioinformatic gene regulatory and protein-protein interaction networks linked to implant success and failure; and the influence of implant surface properties on the molecular response. It then examines how this knowledge may be translated into clinically useful biomarkers and prediction tools. Earlier reviews have described osseointegration either as a clinical and histologic phenomenon [8] or, more recently, in terms of individual signaling pathways [9]. What remains underexplored is an integrated account that links stage-specific gene expression, the principal osteogenic signaling pathways, and network-level bioinformatic evidence within a single translational framework. This review addresses that gap and, in doing so, differs from previous osseointegration reviews by connecting molecular mechanisms to candidate biomarkers and to the emerging goal of patient-specific, implantogenomics-guided care.

This is a narrative review. Relevant English-language records were identified in PubMed/MEDLINE, Scopus, and Web of Science using combinations of the terms osseointegration, dental implant, gene expression, transcriptomics, signaling pathway, bioinformatics, and biomarker. The search covered publications from database inception to April 2026, and the final search was performed in April 2026. Original in vitro, in vivo, and human studies, transcriptomic and bioinformatic analyses, and relevant review articles were considered; records were included when they addressed the molecular, transcriptomic, or network-level determinants of peri-implant bone healing, and were excluded when they reported only mechanical or purely clinical outcomes without molecular data. Reference lists of key articles and publicly available gene expression datasets were also screened. Selected records were critically appraised and synthesized narratively rather than through a formal systematic protocol. Consistent with this design, no formal risk-of-bias instrument was applied, and no quantitative synthesis, such as meta-analysis or meta-regression, was undertaken.

## Review

Biological basis of osseointegration: the cellular cascade from implant placement to bone-implant contact

Osseointegration allows dental implants to achieve long-term functional stability within the host bone. While early investigations focused on the histologic demonstration of bone-to-implant contact, current knowledge recognizes osseointegration as a complex sequence of cellular and molecular events that begins immediately after implant placement and continues throughout the implant's lifespan. Bone healing around implants is regulated by coordinated gene expression programs that control inflammation, cell recruitment, osteogenesis, and remodeling [2,10].

The first biological event occurs when blood comes into contact with the implant surface. Within seconds, plasma proteins adsorb onto the titanium surface, forming a conditioning layer that mediates interactions between the biomaterial and surrounding tissues. Fibronectin and vitronectin are among the most relevant components of this layer because they provide binding sites for integrins expressed on inflammatory and progenitor cells. These early interactions influence cell attachment and activate intracellular signaling pathways that regulate subsequent healing events [10,11].

Acute inflammation follows implant placement and represents a necessary stage of normal wound healing. Neutrophils are rapidly recruited to the surgical site, where they contribute to decontamination and removal of damaged tissue. Macrophages subsequently become the dominant cell population and coordinate tissue repair by secreting cytokines and growth factors. Rather than being purely detrimental, controlled inflammation is now viewed as essential for successful osseointegration because it creates the biological environment required for bone formation. Numerous genes associated with immune regulation and tissue regeneration are activated during this period, generating distinct transcriptional signatures that can be identified through gene expression analyses [2,10].

As the inflammatory response begins to resolve, mesenchymal stem cells migrate toward the implant surface, guided by chemotactic signals released during the early healing phase. Once attached to the protein-coated surface through integrin-mediated mechanisms, these cells undergo osteogenic differentiation and progressively acquire the phenotype of mature osteoblasts. This transition is accompanied by increased expression of genes involved in extracellular matrix synthesis and mineralized tissue formation. Osteoblasts subsequently produce collagen-rich osteoid that serves as the scaffold for new bone deposition and mineralization [2,11].

The newly formed woven bone is gradually replaced by mature lamellar bone through continuous remodeling. This process depends on the balanced activity of osteoblasts and osteoclasts and ultimately determines the quality and durability of the bone-implant interface. Davies described this phenomenon through the bone chamber model, highlighting the dynamic nature of bone formation around endosseous implants and the simultaneous occurrence of contact and distance osteogenesis [12].

Clinically, implant stability reflects the interaction between mechanical and biological factors. Primary stability is achieved at the time of implant placement and depends mainly on implant geometry, insertion torque, and bone density. Secondary stability develops later, resulting from new bone formation and biological fixation. Between these two phases, there is a transitional period during which mechanical stability may decrease before biological stability is fully established. Although this stage is rarely detectable through routine clinical examination, it is characterized by intense molecular activity and substantial changes in gene expression, which is why the intermediate healing phase has become a major focus of transcriptomic and bioinformatic research [2,10]. Implant stability quotient measurements provide useful clinical information about this transition, but gene expression profiling offers a more detailed understanding of the biological mechanisms that ultimately govern implant integration and long-term clinical success.

Key signaling pathways governing osteoblast differentiation at the implant interface: Bone morphogenetic protein (BMP), Wnt/β-catenin (Wnt), Nuclear factor kappa B (NF-κB), and Runt-related transcription factor 2 (RUNX2)

Successful osseointegration depends on coordinated molecular signaling between inflammatory cells, mesenchymal stem cells, osteoblasts, and the implant surface. Transcriptomic and bioinformatic studies indicate that implant integration is controlled not by isolated genes but by interconnected signaling networks that regulate osteogenic differentiation, extracellular matrix formation, angiogenesis, and bone remodeling [13]. Among these networks, the BMP, Wnt/β-catenin, NF-κB, and RUNX2 pathways represent the principal molecular determinants of osteoblast commitment and peri-implant bone formation.

BMP Signaling

BMP signaling is one of the earliest and most potent osteoinductive pathways activated during osseointegration. Following implant placement, BMP-2 and BMP-7 released from surrounding tissues bind to BMP receptors on mesenchymal stem cells and osteoprogenitor cells, inducing phosphorylation of SMAD1, SMAD5, and SMAD8. These proteins form transcriptional complexes that translocate to the nucleus and activate osteogenic genes, including RUNX2 and Osterix (SP7), which are required for osteoblast lineage commitment [14]. BMP signaling serves as a central regulatory node within larger osteogenic networks, influencing genes involved in collagen synthesis, matrix maturation, and mineral deposition, and is consistently associated with successful peri-implant bone formation [15]. A current gap is that much of this evidence derives from in vitro systems and animal models, and the optimal timing and dosing of BMP modulation at the implant surface in humans remain undefined.

Wnt/β-catenin Signaling

The canonical Wnt/β-catenin pathway acts synergistically with BMP signaling to promote osteoblast differentiation and expansion of the osteoprogenitor population. Wnt ligands bind to Frizzled and low-density lipoprotein receptor-related protein 5 and 6 (LRP5/6) receptors, preventing degradation of β-catenin and allowing its accumulation in the cytoplasm. Stabilized β-catenin then enters the nucleus, where it interacts with lymphoid enhancer factor 1 (LEF1) and T-cell factor (TCF) transcription factors to activate osteogenic gene expression, while simultaneously suppressing adipogenic differentiation [16]. Transcriptomic analyses have identified Wnt-associated genes among the most enriched pathways during successful osseointegration [4]. Wnt signaling also regulates the receptor activator of nuclear factor-κB ligand/osteoprotegerin (RANKL/OPG) axis: increased osteoprotegerin production and reduced RANKL expression inhibit osteoclast differentiation, favoring net bone formation around the implant [16]. How Wnt activity might be therapeutically tuned at the implant interface without provoking unwanted systemic skeletal effects is still unresolved and largely untested in humans.

NF-κB Signaling

Unlike the BMP and Wnt pathways, nuclear factor kappa-light-chain-enhancer of activated B cells (NF-κB) signaling has both beneficial and detrimental effects depending on the duration and intensity of activation. During early healing, surgical trauma induces transient NF-κB activation through mediators such as tumor necrosis factor (TNF)-α and IL-1β, promoting immune cell recruitment, angiogenesis, and mesenchymal stem cell migration toward the implant [16]. Prolonged activation, however, shifts the balance toward chronic inflammation, increasing pro-inflammatory cytokines and stimulating osteoclastogenesis through RANKL-dependent mechanisms [17]. Experimental work further shows that sustained NF-κB activity in mesenchymal stem cells can inhibit BMP-2-induced osteoblastic differentiation through the BMP/Smad pathway [18]. Bioinformatic analyses of failed implants frequently identify NF-κB-related genes as central hubs within inflammatory networks, linking them to peri-implant bone loss [19]. The threshold separating beneficial transient activation from harmful sustained signaling has not yet been defined in human peri-implant tissue, thereby limiting its direct clinical use.

RUNX2 as the Master Transcriptional Integrator

RUNX2 functions as the master transcription factor integrating signals from BMP, Wnt, inflammatory mediators, and mechanical stimulation. Its activation induces osteoblast-specific genes including collagen type I alpha 1 chain (COL1A1), alkaline phosphatase, biomineralization associated (ALPL), bone gamma-carboxyglutamate protein (BGLAP or osteocalcin), and SP7, which collectively regulate matrix production, mineralization, and osteoblast maturation. Systems biology approaches consistently identify RUNX2 as a hub gene within osteogenic regulatory networks, linking extracellular signaling to the transcriptional programs responsible for bone formation [2,4]. Importantly, current evidence emphasizes extensive crosstalk among these pathways rather than independent activity: BMP signaling enhances RUNX2 expression, Wnt signaling stabilizes osteogenic transcriptional programs, and controlled NF-κB activation promotes repair, whereas excessive activation disrupts osteogenesis [15]. Most RUNX2 network data, however, come from cross-sectional or preclinical studies, so causal and time-resolved evidence in patients is still lacking.

Transcriptomic profiling of peri-implant tissues across osseointegration stages

Transcriptomic profiling provides a molecular snapshot of which genes are active in a tissue at a given moment, and RNA sequencing now offers a genome-wide view of the transcripts present in healing peri-implant tissue [20]. This helps explain implant healing beyond what can be seen clinically or histologically: not only whether bone-to-implant contact occurs, but which genetic and molecular changes allow that contact to become stable and functional over time [21,22]. Microarrays evaluate previously known sequences, whereas RNA-seq captures a broader range of transcripts, and both can track how peri-implant tissues change through the inflammatory, repair, and remodeling phases [22,23].

During the early inflammatory phase, usually between days one and seven after placement, the tissue responds mainly to surgical trauma. The most active biological processes involve immune response, cytokine signaling, chemokine activity, and cellular recruitment, and inflammatory genes such as IL6, TNF, and C-X-C motif chemokine ligand 8 (CXCL8) are upregulated as part of the initial response that activates tissue repair. This response should be controlled and temporary; if it remains elevated, it may impair integration. Public datasets such as gene expression omnibus (GEO) accession GSE41446, which evaluated human implant-adherent cells at three and seven days, enable transparent, reproducible study of these early changes [24].

During the repair phase, approximately weeks two to four, the process shifts toward extracellular matrix organization, angiogenesis, collagen deposition, and osteogenic differentiation. Genes such as COL1A1 and vascular endothelial growth factor A (VEGFA) become clinically meaningful: COL1A1 provides structural support for new tissue, and VEGFA supports the vascularization needed to deliver oxygen and nutrients to the healing area. Studies on nanoscale surfaces and nano-hydroxyapatite coatings suggest that surface modifications can favor the expression of bone-formation genes and early osteogenic activity [21,25].

In the remodeling phase, which may extend from months two to six, the transcriptomic signature becomes more closely associated with mineralization, osteocyte maturation, and long-term remodeling. Markers such as ALPL and BGLAP indicate progression toward mature mineralized tissue, while inflammatory genes such as IL6, TNF, and CXCL8 should decline as healing stabilizes. Persistent expression of these inflammatory genes may shift the molecular environment toward peri-implant inflammation or failure [22,23].

Human studies also show that peri-implant tissue is not biologically identical to periodontal gingiva. Genes such as secreted phosphoprotein 1 (SPP1), cystatin SN (CST1), aquaporin 9 (AQP9), and secreted Frizzled-related protein 2 (SFRP2) have been reported to be upregulated in peri-implant soft tissue, suggesting that the soft tissue seal around implants has its own molecular identity [26]. Datasets such as GSE223924 may be useful for comparison with peri-implant disease, although they should not be interpreted as a direct longitudinal record of osseointegration [27]. Methodologically, RNA-seq data can be analyzed with tools such as DESeq2 [28] or edgeR [29], and microarray datasets with GEO2R, while repositories such as GEO [30] and ArrayExpress [31] support validation and reproducibility; genome-wide transcriptional responses of osteoblasts to different titanium surface topographies have been characterized using these approaches [32]. Overall, transcriptomic profiling connects the biological stages of healing with clinically relevant outcomes and provides the foundation for network-level analysis of hub genes and regulatory pathways. It should be noted that most of this evidence comes from animal models, small human cohorts, and heterogeneous platforms, so the reported signatures require confirmation in larger standardized studies.

Bioinformatic analysis of gene regulatory networks and protein-protein interaction (PPI) hubs associated with implant success and failure

Bioinformatic approaches analyze interactions among many genes and proteins rather than evaluating individual genes in isolation, thereby enabling the identification of regulatory networks associated with implant success or failure [2,19]. PPI analysis is particularly useful for detecting hub genes, the highly connected molecules that play critical biological roles. The analysis typically begins with differentially expressed genes from transcriptomic studies, which are imported into the search tool for the retrieval of interacting genes/proteins (STRING) database to construct PPI networks, then analyzed in Cytoscape using the CytoHubba plugin. Hub genes are identified using topological parameters such as degree, betweenness centrality, and closeness centrality, and functional enrichment is assessed using Gene Ontology and Kyoto encyclopedia of genes and genomes (KEGG) databases to determine the biological pathways represented within these networks [19].

Studies of successful osseointegration consistently identify hub genes involved in osteogenesis, angiogenesis, and tissue regeneration. Among the most relevant are AKT serine/threonine kinase 1 (AKT1), VEGFA, tumor protein 53 (TP53), and RUNX2: AKT1 regulates cell survival and proliferation, VEGFA promotes vascularization, TP53 contributes to cellular homeostasis, and RUNX2 controls osteoblast differentiation. Enrichment analyses commonly reveal pathways related to phosphoinositide 3-kinase (PI3K)-AKT signaling, extracellular matrix organization, and bone formation, supporting their importance in implant integration [4,10].

Failed implant networks, by contrast, are characterized by increased expression of inflammatory and osteoclastogenic genes. Important hubs include IL6, TNF, matrix metalloproteinase-9 (MMP9), and nuclear factor of kappa light polypeptide gene enhancer in B-cells 1 (NFKB1), which are associated with chronic inflammation, matrix degradation, and excessive bone resorption, and enrichment analyses frequently demonstrate activation of NF-κB signaling and cytokine-mediated inflammatory pathways. Compared with successful osseointegration, these networks show reduced osteogenic activity and greater inflammatory connectivity [16,19].

Recent studies have incorporated microRNA regulation into network analysis. MicroRNAs act as post-transcriptional regulators that influence multiple targets simultaneously; miR-21 has been associated with enhanced osteoblast differentiation and bone formation, whereas miR-34a participates in regulating osteoclast activity and bone remodeling. Platforms such as miRNet and GeneMANIA integrate these regulatory layers with traditional PPI networks [4,19]. Together, the integration of differential expression analysis, PPI networks, hub gene identification, and pathway enrichment improves understanding of osseointegration and may contribute to the identification of predictive biomarkers and personalized implant therapies. The recurring genes, pathways, and network hubs associated with successful and failed integration across these healing phases are summarized in Table 1.

**Table 1:** Key genes, signaling pathways, and network hubs associated with implant success and failure across the phases of osseointegration BMP: bone morphogenetic protein; PPI: protein-protein interaction; RANKL: receptor activator of nuclear factor-κB ligand (TNFSF11); OPG: osteoprotegerin (TNFRSF11B); VEGF: vascular endothelial growth factor; COL1A1: collagen type I alpha 1 chain; ALPL: alkaline phosphatase, biomineralization associated; BGLAP: bone gamma-carboxyglutamate protein; PIK3-AKT: phosphoinositide 3-kinase-AKT serine/threonine kinase 1; TP53: tumor protein. [4,10-19]

Functional category	Representative genes	Associated pathway(s)	Healing phase	Network role and clinical association
Osteoblast commitment and differentiation	RUNX2, SP7 (Osterix), ALPL, BGLAP, COL1A1	BMP/Smad; Wnt/β-catenin	Repair to remodeling	Recurring osteogenic hub genes in successful integration; drive matrix production and mineralization
Angiogenesis	VEGFA	PI3K-AKT; VEGF	Repair	Supports vascularization of healing tissue; enriched in successful osseointegration
Cell survival and proliferation	AKT1, TP53	PI3K-AKT	Repair	High-connectivity hubs in success networks; regulate progenitor survival and homeostasis
Early physiologic inflammation	IL6, TNF, CXCL8, IL1B	NF-κB; cytokine signaling	Inflammatory (days 1 to 7)	Necessary when transient; initiates repair, but sustained expression predicts poor integration
Chronic inflammation and resorption (failure)	IL6, TNF, MMP9, NFKB1	NF-κB; cytokine-mediated inflammation	Sustained or late	Dominant hubs in failed-implant networks; matrix degradation and excessive bone resorption
Remodeling balance	TNFSF11 (RANKL), TNFRSF11B (OPG)	RANKL/OPG; Wnt	Remodeling	RANKL/OPG ratio governs osteoclast activity; shifts favor formation or resorption
Post-transcriptional regulation	miR-21 (pro-osteogenic), miR-34a (osteoclast regulation)	microRNA network layer	Repair to remodeling	Modulate multiple targets; integrated with PPI networks via miRNet and GeneMANIA

Influence of implant surface properties on the molecular response

Implant surface engineering regulates the molecular events at the bone-implant interface by altering protein adsorption, cell adhesion, immune signaling, and osteogenic differentiation. Current evidence indicates that surface topography, hydrophilicity, and material composition directly affect the transcriptional programs associated with successful osseointegration.

Surface roughness is a critical determinant of cellular behavior during early healing. Compared with machined titanium, micro- and nano-rough surfaces generated through sandblasting, acid etching, or anodization enhance osteoblast attachment by promoting integrin clustering and focal adhesion formation. Integrin β1 (ITGB1) acts as a primary mechanosensor at the cell-implant interface, initiating signaling through focal adhesion kinase (PTK2/FAK) and recruitment of cytoskeletal proteins such as vinculin (VCL). Activation of these pathways stimulates osteogenic differentiation through downstream mitogen-activated protein kinase/extracellular signal-regulated kinase (MAPK/ERK) and Wnt/β-catenin signaling, and roughened titanium surfaces induce increased expression of adhesion-related and osteogenic genes, including RUNX2, COL1A1, and BGLAP, relative to smoother surfaces, indicating that topography regulates transcriptional activity rather than merely improving mechanical retention [33,34]. In vivo transcriptomic analyses further show that microrough titanium surfaces modulate gene expression associated with bone regeneration, extracellular matrix formation, and tissue remodeling during healing [35].

Surface wettability also modulates the molecular response. Hydrophilic surfaces such as chemically modified hydrophilic SLA surface (SLActive) implants exhibit lower contact angles and greater surface energy than conventional sandblasted, large-grit, acid-etched (SLA) surfaces, facilitating rapid adsorption of matrix proteins, particularly fibronectin, and accelerating early cell attachment. A human transcriptomic study comparing SLA and SLActive implants demonstrated earlier activation of osteogenic and angiogenic gene programs, with BMP- and VEGF-associated genes upregulated on SLActive surfaces after seven days of healing [36]. Hydrophilic surfaces also influence innate immunity: experimental work shows that they promote macrophage polarization toward a regenerative M2 phenotype, with increased mannose receptor C-type 1 (MRC1), arginase-1 (ARG1), and IL10, and reduced TNF, IL1B, and IL6, thereby creating an osteoimmunomodulatory environment favorable for osteoblast recruitment, angiogenesis, and matrix deposition [37].

Material composition is a further determinant. Commercially pure titanium and titanium alloys remain the standard because of their biocompatibility and a stable titanium oxide layer that supports osteoblast adhesion, while zirconia has emerged as an esthetic alternative with favorable soft tissue integration. Comparative transcriptional analyses of osteoblasts on titanium and zirconia show that both substrates activate genes involved in tissue regeneration, matrix remodeling, and osteogenic differentiation; in an evaluation of 90 tissue-integration genes, biomaterial chemistry influenced expression patterns more than surface topography alone, regulating ITGB1, RUNX2, SP7, matrix collagens, BGLAP, and bone-regeneration growth factors [38]. Additional comparisons report similar activation of adhesion and osteogenic markers, with appropriately engineered zirconia surfaces achieving responses comparable to those of titanium and to surface modifications that increase roughness or bioactivity, further enhancing RUNX2, COL1A1, and BGLAP expression [39]. Collectively, these findings establish a direct mechanistic link between implant surface design and the molecular determinants of successful osseointegration (Table 2).

**Table 2:** Influence of implant surface characteristics on hydrophilicity, molecular pathways, gene expression, and biological response in osseointegration ITGB1: integrin beta1; PTK2: protein tyrosine kinase 2 (focal adhesion kinase, FAK); VCL: vinculin; RUNX2: runt-related transcription factor 2; COL1A1: collagen type I alpha 1 chain; BGLAP: osteocalcin; MRC1: mannose receptor C-type 1; SLA: sandblasted, large-grit, acid-etched; SLActive: chemically modified hydrophilic SLA surface. [35-37,39]

Surface type	Surface characteristics	Hydrophilicity	Principal molecular pathway	Key genes upregulated	Primary biological effect
Machined titanium	Smooth surface, low roughness	Low	Weak focal adhesion signaling	ITGB1, PTK2, RUNX2 (baseline)	Reduced osteoblast differentiation
SLA	Moderately rough microtopography	Moderate	Integrin beta1 to FAK mechanotransduction	ITGB1, PTK2, RUNX2, COL1A1	Enhanced cell adhesion and matrix formation
SLActive	Rough microtopography, high surface energy	High	Fibronectin adsorption, osteoimmunomodulation	RUNX2, COL1A1, BGLAP, MRC1	Accelerated osseointegration and M2 polarization
Anodized titanium	Micro/nanostructured oxide layer	Moderate	FAK activation and cytoskeletal signaling	PTK2, VCL, COL1A1	Enhanced mineralization and bone formation
Surface-modified zirconia	Variable roughness depending on treatment	Variable	Adhesion and osteogenic differentiation pathways	ITGB1, RUNX2, COL1A1, BGLAP	Osteogenic response comparable to titanium

Translational potential: gene expression-based biomarkers for predicting osseointegration outcomes

The molecular determinants reviewed above are clinically meaningful only if they can be measured and acted upon. Three translational directions are beginning to define how transcriptomic and bioinformatic knowledge may enter prosthodontic practice.

The first concerns biomarkers that can be sampled without disturbing the healing interface. Peri-implant crevicular fluid (PICF) and gingival crevicular fluid contain proteins whose genes are prominent in the networks described earlier, and several of these can be quantified with chairside immunoassays. Systematic reviews and meta-analyses report that interleukin-1β and matrix metalloproteinase-8 are consistently elevated around diseased implants, while the soluble RANKL/OPG ratio and vascular endothelial growth factor show moderate predictive value for peri-implant breakdown [40,41]. Comparative analyses of RANKL and OPG in PICF support a biological link between these molecules and the remodeling balance governing secondary stability [42]. At present, these markers describe established disease more reliably than they predict it, and validated diagnostic thresholds are still lacking [40].

The second direction is patient-specific risk profiling. Because osseointegration depends on immune-inflammatory and osteogenic gene programs, inherited variation in those genes may help explain why some patients integrate implants poorly despite adequate surgery. Candidate variants discussed in the implantogenomics literature include polymorphisms in the IL-1 gene cluster, TNF-α, and the RANKL/OPG axis, although reported associations remain inconsistent and require larger, well-controlled cohorts before they can be used to stratify patients prior to loading [4,16]. Altered molecular signatures in systemic conditions such as type II diabetes illustrate how host biology reshapes the peri-implant transcriptome and, by extension, individual risk [43].

The third direction is precision implantology. Integrating transcriptomic, proteomic, and clinical data through machine learning pipelines could, in principle, match implant surface, material, and loading protocol to a patient's molecular profile, and early work combining bioinformatics with machine learning in peri-implant research points in this direction [19]. Genome-wide reviews of implant integration similarly argue that multidimensional, cross-cellular data are needed to explain why promising laboratory surfaces do not always succeed in vivo [20].

Progress toward clinical adoption depends on closing several gaps. Much of the current transcriptomic and bioinformatic evidence derives from in vitro systems and animal models; sample sizes are small; and sampling and analysis methods differ across studies, limiting comparability and reproducibility. Sampling and analysis of PICF must therefore be standardized so results are comparable across studies. Prospective longitudinal studies are needed to map gene expression against documented integration outcomes rather than against established disease. And prediction models must be validated in independent cohorts with reported sensitivity and specificity before they can guide treatment. Addressing these priorities would move the emerging field of implantogenomics from descriptive association toward genuinely personalized implant rehabilitation [4,16].

## Conclusions

Osseointegration is best understood as a coordinated molecular program rather than a purely mechanical event. The cellular cascade that follows implant placement is driven by interconnected BMP, Wnt/β-catenin, NF-κB, and RUNX2 signaling and produces stage-specific transcriptomic signatures that shift from immune activation toward matrix formation and mineralization. Bioinformatic network analyses repeatedly identify osteogenic and angiogenic hubs in successful integration and inflammatory, osteoclastogenic hubs in failure, and implant surface topography, hydrophilicity, and composition measurably shape these programs. Several limitations temper these conclusions. Much of the supporting evidence comes from in vitro systems and animal models; cohorts are small, and sampling and analysis methods differ across studies, limiting comparability. Standardized methodologies, larger clinical validation cohorts, and longitudinal human studies that map gene expression against documented integration outcomes are therefore needed before molecular biomarkers can enter routine clinical practice. Translating this knowledge into care will depend on non-invasive biomarkers, genetically informed risk profiling, and validated computational prediction. None of these tools is ready for routine use today, but together they outline a credible path toward an implantogenomics-guided, patient-specific approach to implant-supported prosthetic rehabilitation.
